# Retrospective Evaluation of the Epidemiology and Practice Variation of Dexmedetomidine Use in Invasively Ventilated Pediatric Intensive Care Admissions, 2007–2013

**DOI:** 10.3389/fped.2015.00109

**Published:** 2015-12-16

**Authors:** Brian D. Benneyworth, Stephen M. Downs, Mara Nitu

**Affiliations:** ^1^Section of Pediatric Critical Care Medicine, Department of Pediatrics, Indiana University School of Medicine, Indianapolis, IN, USA; ^2^Indiana Children’s Heath Services Research, Department of Pediatrics, Indiana University School of Medicine, Indianapolis, IN, USA

**Keywords:** physician’s practice patterns, intensive care units, pediatric, dexmedetomidine, diagnosis-related groups, drug utilization, respiration, artificial

## Abstract

**Objectives:**

The study assessed dexmedetomidine utilization and practice variation over time in ventilated pediatric intensive care unit (PICU) patients; and evaluated differences in hospital outcomes between high- and low-dexmedetomidine utilization hospitals.

**Study design:**

This serial cross-sectional analysis used administrative data from PICU admissions in the pediatric health information system (37 US tertiary care pediatric hospitals). Included admissions from 2007 to 2013 had simultaneous dexmedetomidine and invasive mechanical ventilation charges, <18 years of age, excluding neonates. Patient and hospital characteristics were compared as well as hospital-level severity-adjusted indexed length of stay (LOS), charges, and mortality.

**Results:**

The utilization of dexmedetomidine increased from 6.2 to 38.2 per 100 ventilated PICU patients among pediatric hospitals. Utilization ranged from 3.8 to 62.8 per 100 in 2013. Few differences in patient demographics and no differences in hospital-level volume/severity of illness measures between high- and low-utilization hospitals occurred. No differences in hospital-level, severity-adjusted indexed outcomes (LOS, charges, and mortality) were found.

**Conclusion:**

Wide practice variation in utilization of dexmedetomidine for ventilated PICU patients existed even as use has increased sixfold. Higher utilization was not associated with increased hospital charges or reduced hospital LOS. Further work should define the expected outcome benefits of dexmedetomidine and its appropriate use.

## Introduction

Dexmedetomidine is a centrally acting alpha-2 adrenergic receptor agonist that was licensed by the Food and Drug Administration for use in adult patients in the intensive care unit and perioperative areas in 1999. Initial adult and animal studies were conducted during the 1990s and early 2000s. Since that time the number of human reports indexed by Medline has dramatically increased. Pediatric reports began in 2002; and, since 2008, there have been approximately 50 published articles annually.

Numerous benefits of dexmedetomidine in pediatrics have been reported, but they are largely based on physiology evaluated in animals and observational human studies. Randomized control trials are mostly limited to the peri-operative period. Dexmedetomidine’s reported benefits include (1) maintenance of spontaneous breathing in sedated patients, (2) potential neuroprotective qualities, (3) reduced need for/tolerance to other sedative agents, (4) sedative/heart rate control in cardiac surgical patients, (5) facilitating tracheal extubation, and (6) reduced delirium in the postoperative/prolonged sedation states (1–7).

As overall mortality is low in pediatric critical care, most research focuses on short-term, surrogate outcomes, such as length of stay (LOS) or duration of mechanical ventilation. Dexmedetomidine has demonstrated an ability to reduce opioid and benzodiazepine use as well as attenuating delirium in children (8–10). These benefits have been hypothesized to shorten the critical illness course, but this has not been demonstrated consistently. Long-term studies regarding cognition and neuro-development have not been performed on any sedative regimen. Dexmedetomidine, until coming off patent in 2014, has been approximately 12-fold more expensive than midazolam based on average wholesale pricing (11). Advocates argue that the cost differences are balanced by dexmedetomidine’s benefits, but this assertion has not been evaluated.

In 2012, the Institute of Medicine released its report *The Best Care At Lower Cost* (12) describing the model of a learning health care system. The report, in part, focuses on applying the appropriate knowledgebase in real time to patient care and emphasizes the vast amount of unused clinical data available to researchers and clinicians. It also addressed the limitations of the traditional models of health care research that focus on the randomized control trial. Few randomized control trials exist for dexmedetomidine, and the numerous observational studies inconsistently suggest a variety of benefits. Without clear recommendation for dexmedetomidine’s clinical use, physicians are left to apply their individual judgment.

This study hypothesizes that the utilization and variation have increased over time without clear evidence of benefit in outcomes related to healthcare utilization. It aims to define the time trends in utilization of dexmedetomidine in pediatric intensive care unit (PICU) patients who require invasive mechanical ventilation by analyzing current, readily available administrative data. It subsequently aims to use the naturally occurring practice variation between high- and low-dexmedetomidine utilization hospitals to evaluate the difference in aggregated hospital-level outcomes (hospital charges, LOS, and mortality) for all mechanically ventilated PICU patients.

## Materials and Methods

### Study Design and Dataset

This was a retrospective, serial cross-sectional study of PICU admissions requiring invasive mechanical ventilation among hospitals reporting to the pediatric health information system (PHIS). PHIS is an administrative database that contains inpatient, emergency department, and ambulatory surgery data from 47 not-for-profit, tertiary care pediatric hospitals with teaching services in the United States. The hospitals are affiliated with the Children’s Hospital Association (CHA; Shawnee Mission, KS, USA), a business alliance of children’s hospitals. Data quality and reliability are assured through a joint effort between the CHA and participating hospitals. The data warehouse function for the PHIS database is managed by Thomson Reuters (Ann Arbor, MI, USA). Data are de-identified at the time of data submission, and data are subjected to a number of reliability and validity checks before being included in the database. The PHIS dataset contains revenue codes, which are mapped to the clinical transaction classification (CTC) system. This permits more detailed evaluation of billed services during a hospitalization (13). Ten hospitals that either did not submit revenue code information or had significant data quality issues were excluded from analysis. PHIS hospitals are commonly referred to as freestanding because they are required to be geographically distinct institutions. The formal definition of a freestanding children’s hospital also requires that it be self-governing and in a separate facility than an affiliated adult hospital. Because many large pediatric hospitals exist within the context of larger academic health systems, some PHIS hospitals are actually pediatric units within a general hospital.

The Institutional Review Board of Indiana University approved this study and waived the requirement of informed consent.

### Identification of Sample

This analysis was limited to inpatients admitted between January 1, 2007 and December 31, 2013 from 37 PHIS hospitals. As hospital discharge after critical illness can be delayed, data analysis and results were reported in terms of hospital admission date in order to group care that occurred in similar time periods. Since administrative data are captured at the time of discharge, 2013 admissions were included if discharge occurred by December 31, 2014 (the most recent PHIS data load). Admissions were included if the patient was <18 years of age and had the PHIS ICU flags (Data Sheet S1 in Supplementary Material). Dexmedetomidine use in patients who were not invasively ventilated (non-invasive positive pressure ventilation or no respiratory support) was excluded from this analysis. Neonatal admissions with Neonatal ICU flag or All-Patient Refined Diagnosis Related Group (3M Health Information Systems, APR DRG) version 30 codes related to neonatal care were excluded (Data Sheet S1 in Supplementary Material).

### Analysis of Hospital-Level Utilization and Outcomes

Utilization of dexmedetomidine was defined as the presence of a CTC pharmacy code for dexmedetomidine (114055) occurring on the same day of service as a CTC service code for mechanical ventilation (521166, 521169). Only admissions with an intensive care unit service charge were included. CTC codes reflect care that was ordered; administration and dosing cannot be verified. Utilization of dexmedetomidine over time was expressed as the rate of admissions with dexmedetomidine CTC code per 100 invasively ventilated PICU admissions. Utilization was evaluated in 6-month time intervals based on hospital admission date.

Characteristics of mechanically ventilated PICU patients were aggregated at the hospital-level to allow comparisons among hospitals with different levels of dexmedetomidine utilization. These characteristics include age groups (<1, 1–4, 5–13, and ≥13 years), gender, race/ethnicity (Caucasian, African-American, and Hispanic), and primary payer (public and private). Dexmedetomidine use in patients with complex chronic conditions was compared (14). Since dexmedetomidine has potential benefits in (1) cardiac surgical patients, (2) neurosurgical/neurological care (including neuro-trauma), and (3) respiratory disease with prolonged invasive ventilation, these groups were analyzed separately based on APR DRG codes (Data Sheet S1 in Supplementary Material).

Hospital characteristics were aggregated to facilitate comparisons. Dexmedetomidine utilization, as measured in 2012 and 2013, was grouped into tertiles representing high, intermediate, and low utilization. The effects of PICU volume and severity of illness (SOI) on dexmedetomidine utilization was assessed. The PICU flag defined the total number of annual PICU admissions; and the cardiac surgical volume, defined as the sum of the risk adjustment in congenital heart surgery, version 1 (RACHS-1), categories 1–6 for all patients admits to the hospital (15). To evaluate SOI the proportion of PICU patients who required invasive mechanical ventilation and the proportion of PICU patients in each APR DRG SOI category 3 and 4 were determined for each hospital. SOI categories 3 and 4 are the most severely ill patients.

Hospital-level outcomes for pediatric patients requiring the PICU admission and mechanical ventilation were severity-adjusted LOS, charge, and mortality indexes. These indexes were calculated based on the total observed LOS (or charges/mortality) for the population of invasively ventilated PICU patients divided by the total severity-adjusted expected LOS (or charges/mortality) at each hospital. NICU admissions were not excluded for cardiac surgical patients, as many have encounters in both units. The severity adjustment was determined from the APR DRG weighting structure defined by 3M and Truven (16). Each APR DRG has four corresponding SOI levels that are coded based on diagnoses, procedures, and demographics. There are specified weights for LOS, charges, and mortality for each APR DRG and severity level. Adjusted hospital charges were used for the observed charges, which accounts for differences in value of medical services in different economic markets. Severity-adjusted LOS, charge, and mortality indexes were also determined for each diagnostic group to look for differences in specific subpopulations. The use of APR DRG severity adjustment in pediatric and intensive care literature has been discussed (17–20).

### Statistical Analysis

All analyses were completed with Stata 12.1 (Stata Corp., College Station, TX, USA).

*The change in utilization of dexmedetomidine* from January 2007 through December 2013 was evaluated by comparing the median rate of utilization, interquartile range (IQR), and full range for each 6-month time period for available hospitals. The trend in utilization over time was evaluated using variance-weighted least squares regression (21). Because of an obvious change in the growth of utilization occurring in 2011 or 2012, a two-part regression model was used. Only 3 hospitals (of the 37) did not have complete utilization data going back to 2007, but they all contributed at least 5 years of data.

*To compare outcomes by dexmedetomidine utilization*, dexmedetomidine utilization was characterized in the 2012 and 2013 admissions. Hospitals were grouped by their utilization patterns as previously discussed. In each hospital, the proportions of each patient characteristic for those receiving dexmedetomidine were determined. To evaluate for a potential effect of dexmedetomidine the median proportion and IQR across the hospitals in the low- and high-utilization groups were then compared using ranksum testing. Aggregate hospital characteristics were similarly evaluated. Severity-adjusted indexed outcomes were compared using ranksum and presented as median with IQR. The intermediate utilization group was presented for comparison purposes only. Statistical significance was set at a *p* < 0.05.

## Results

### Trends in Variation and Utilization in Dexmedetomidine, 2007–2013

**Figure 1:**
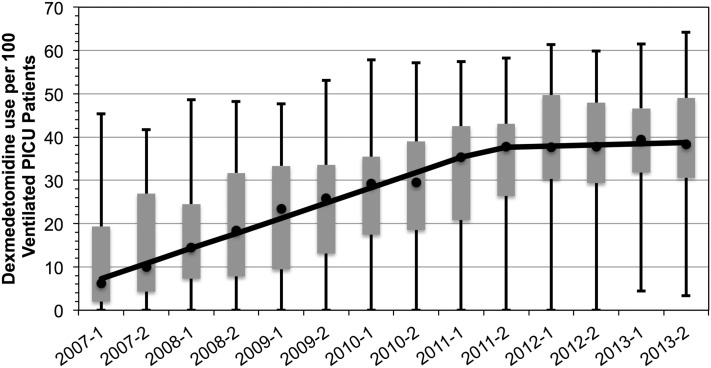
**Dexmedetomidine utilization in ventilated pediatric intensive care patients, 2007–2013**. Rate of utilization per 100 invasively ventilated PICU patients in 6-month time intervals. Markers reflect the median rate of utilization with the IQR as the gray box. Full range denoted by the wiskers. Median rate of increase overtime is denoted by the solid line and indicates a rate of increase of 7 PICU patients per 100 each year until 2012 (*p* = 0.02) after which it remains constant.

Median utilization of dexmedetomidine across the 37 pediatric hospitals increased from 6.2 to 38.3 per 100 mechanically ventilated PICU admissions (an over sixfold increase in 7 years, Figure 1). There was a statistically significant increase in utilization from 2007 through 2012; the utilization was constant in 2012 and 2013. Despite the expected increase in utilization, practice variation persisted. The size of the IQR did not decrease; and furthermore, in 2013, the full range of utilization was 3.8 to 62.8 per 100 mechanically ventilated PICU patients.

### Patient Characteristics in High- and Low-Utilization Hospitals, 2012–2013

While the median rate of dexmedetomidine utilization was statistically different in low- vs. high-utilization hospitals (23.7 vs. 50.6 per 100 mechanically ventilated PICU admissions), there were only nominal differences in distribution of patient age, gender, race/ethnicity, and primary payers for those who received dexmedetomidine (Table 1). There was a statistically significant lower median proportion of toddlers 1–4 years in high-utilization hospitals, with a likely increased proportion of children <1 year of age. Regardless of the level of dexmedetomidine utilization in a particular hospital, approximately 50–55% of mechanically ventilated patients who received dexmedetomidine were admitted for conditions that have been reported to benefit from dexmedetomidine (e.g., cardiovascular surgery, neurosurgery/neurological care, and prolonged mechanical ventilation). There were no differences in pattern of utilization in these disease states based on the level of dexmedetomidine utilization at the hospital.

**Table 1:** **Patient characteristics among intermediate, high, and low utilization hospitals, 2012–2013**.

	Low utilization		Intermediate utilization		High utilization	
	Median %	IQR	Median %	IQR	Median %	IQR
Group’s median utilization rate	23.7		39.7		50.6	
Female gender	43.3	(39.7, 45.0)	43.5	(41.2, 45.2)	42.9	(41.7, 45.3)^NS^
Age groups						
0–1 year	35.6	(29.4, 42.7)	39.3	(32.8, 43.0)	41.0	(38.8, 43.1)^NS^
1–4 years	33.4	(30.3, 36.3)	29.0	(28.1, 32.4)	28.8	(26.7, 29.8)^1^
5–13 years	16.4	(14.6, 20.0)	19.0	(17.4, 23.0)	18.8	(17.6, 20.1)^NS^
≥13 years	12.3	(9.7, 13.6)	10.9	(9.5, 13.1)	12.0	(10.7, 13.5)^NS^
Race categories						
Caucasian	48.2	(37.0, 57.2)	49.8	(39.0, 70.9)	54.4	(46.3, 62.9)^NS^
African-American	17.9	(3.4, 23.9)	16.2	(9.1, 28.2)	15.1	(7.0, 20.0)^NS^
Hispanic	11.9	(7.7, 23.4)	9.2	(4.6, 25.8)	11.9	(2.0, 32.3)^NS^
Payer categories						
Private payer	36.2	(28.1, 44.7)	38.5	(33.0, 43.9)	34.1	(31.1, 42.1)^NS^
Public payer	62.0	(51.9, 68.8)	56.3	(53.9, 63.7)	62.0	(55.5, 64.6)^NS^
Disease states						
Complex chronic illness	83.3	(77.1, 84.7)	86.0	(78.9, 88.2)	82.4	(79.8, 86.1)^NS^
Cardiovascular surgery	29.9	(19.7, 41.7)	34.7	(25.1, 41.6)	30.4	(27.9, 41.3)^NS^
Neurosurgical/neurological care	7.4	(6.2, 10.3)	7.7	(5.0, 10.3)	6.7	(4.9, 11.4)^NS^
Prolonged mechanical ventilation	18.3	(10.9, 20.7)	13.3	(11.6, 15.8)	13.5	(11.6, 16.0)^NS^

*Median percent of patients in each characteristic among mechanically ventilated PICU patients simultaneously billed for mechanical ventilation and dexmedetomidine*.

*Statistical difference between high- and low-utilization groups: NS, not significant (*p* > 0.05), ^1^*p* < 0.01*.

### Characteristics of Hospitals in High and Low-Utilizing PICUs, 2012–2013

In general, hospitals in the high- and low-utilization groups were not different. They had similar numbers of overall PICU admissions, cardiac surgical volume, and SOI measures (proportions of patients requiring mechanical ventilation and APR DRG SOI categories 3 and 4) (Table 2).

**Table 2:** **Hospital characteristics among intermediate, high, and low utilization hospitals**.

	Low utilization		Intermediate utilization		High utilization	
	Median	IQR	Median	IQR	Median	IQR
Volume measures (*n*)						
Annual PICU admissions	1582	(977, 2602)	2283	(1678, 2657)	1614	(1475, 2241)^NS^
Annual cardiac surgical volume	184	(116, 340)	233	(145, 314)	224	(180, 249)^NS^
PICU severity of illness measures (%)						
Mechanical ventilation admits	36.8%	(26.9, 53.4)	43.9%	(37.5, 46.9)	36.9%	(31.3, 45.2)^NS^
APR DRG SOI category 3	32.4%	(28.7, 34.3)	31.7%	(29.0, 33.7)	30.5%	(29.7, 34.0)^NS^
APR DRG SOI category 4	20.3%	(15.2, 22.2)	24.2%	(17.4, 27.2)	20.7%	(19.1, 21.9)^NS^

*Statistical difference between high- and low-utilization groups: NS, not significant (*p* > 0.05)*.

### Hospital Outcomes for High and Low Utilizers of Dexmedetomidine, 2012–2013

Severity-adjusted LOS, charges, and mortality as defined by the observed vs. expected ratio for all mechanically ventilated PICU patients were not different in low and high-utilizing hospitals. The lack of difference persisted even when evaluating the subgroups of disease categories (cardiovascular surgery, neurosurgical/neurological care, or prolonged mechanical ventilation) (Table 3).

**Table 3:** **Indexed hospital-level outcomes among intermediate, high, and low utilization hospitals**.

	Low utilization		Intermediate utilization		High utilization	
	Median	IQR	Median	IQR	Median	IQR
Severity-adjusted observed vs. expected LOS						
Overall	1.29	(1.11, 1.52)	1.19	(1.07, 1.42)	1.28	(1.18, 1.46)^NS^
Cardiovascular surgery	1.18	(1.07, 1.51)	1.13	(1.10, 1.29)	1.11	(1.01, 1.24)^NS^
Neurosurgical/neurological care	1.19	(1.00, 1.32)	1.01	(0.93, 1.59)	1.37	(1.16, 1.60)^NS^
Prolonged mechanical ventilation	1.17	(1.07, 1.45)	1.12	(0.96, 1.43)	1.27	(1.13, 1.42)^NS^
Severity-adjusted observed vs. expected charges						
Overall	1.73	(1.15, 2.30)	1.85	(1.40, 2.00)	1.61	(1.46, 1.97)^NS^
Cardiovascular surgery	1.45	(1.25, 2.39)	1.68	(1.29, 1.85)	1.64	(1.44, 1.72)^NS^
Neurosurgical/neurological care	1.62	(1.12, 1.80)	1.65	(1.46, 1.98)	1.62	(1.51, 1.97)^NS^
Prolonged mechanical ventilation	1.54	(0.89, 2.32)	1.73	(1.20, 2.13)	1.44	(1.36, 1.74)^NS^
Severity-adjusted observed vs. expected mortality						
Overall	0.83	(0.70, 1.04)	0.81	(0.71, 0.95)	0.83	(0.71, 0.88)^NS^
Cardiovascular surgery	0.82	(0.59, 0.90)	0.68	(0.47, 0.92)	0.84	(0.64, 0.89)^NS^
Neurosurgical/neurological care	0.81	(0.72, 1.06)	0.86	(0.58, 0.96)	0.77	(0.55, 0.91)^NS^
Prolonged mechanical ventilation	0.93	(0.65, 1.05)	0.85	(0.70, 0.96)	0.85	(0.75, 1.00)^NS^

*Statistical difference between high- and low-utilization groups: NS, not significant (*p* > 0.05)*.

## Discussion

This analysis presented the natural history of the rise in utilization of dexmedetomidine in invasively ventilated PICU patients in large pediatric hospitals following its introduction and published use. The increase in utilization was not surprising given that practitioners will naturally increase their usage of a potentially beneficial medication, especially when published literature may support the practice. This analysis also demonstrated that as utilization increases practice variation persists. While one might have expected that all hospitals would have increased their use similarly, this analysis indicated that some hospitals continue to have very little use of dexmedetomidine despite the growing trend.

This type of practice variation is ubiquitous in medical care even when there is evidence for effective care (22), including pediatric critical care (23, 24). Because most critical care therapies are not rigorously evaluated, their use is a product of supply sensitive and physician preference-sensitive care. Supply sensitive care should be driven by the optimal rate of utilization; as excess utilization has been found to be harmful (25, 26). Preference-sensitive care is traditionally balanced by the patient’s preferences in regards to therapies and outcomes, which is not possible in most critical care decisions, so physicians invoke their own practice styles (22, 27). The differences in practice style can increase costs without improving outcomes for patients (27).

Differences in patient demographics between high- and low-utilization hospitals did not account for differences in utilization. PICU volume and SOI did not account for differences in utilization, either. It appeared that about half of the use of dexmedetomidine in ventilated PICU patients occurred in disease states with suggested benefit. Current research may support its use in these populations, but the extrapolation to other populations may contribute to practice variation.

Dexmedetomidine has been touted to improve outcomes by reducing the negative effects of opioids and benzodiazepines, facilitating tracheal extubation, and potentially shortening the duration of mechanical ventilation (2–9). A reduction in mechanical ventilation duration may shorten hospital LOS. If a reduction in PICU therapies, such as mechanical ventilation, or overall hospital LOS can translate into reduced healthcare expenditures that offset the cost of dexmedetomidine then there is a substantial benefit. Because many factors contribute to LOS, this study was not designed to compare individual patients who received dexmedetomidine. Instead, this study assessed whether hospitals that had high use of dexmedetomidine had shorter observed vs. expected LOS (indexed LOS) than low-utilization hospitals. This methodology potentially biases the analysis toward finding a reduction. Because high-utilization hospitals use dexmedetomidine in more patients, increased utilization should have a greater effect on the indexed LOS. No reduction in severity-adjusted indexed LOS was found. Because of the added expense of dexmedetomidine, a greater indexed charge ratio in higher utilization hospitals might have been expected, but this difference was not found. No mortality benefit has been suggested with dexmedetomidine in previous studies, nor was one found in this study even with the relatively large sample size. While this severity-adjustment methodology is imperfect it likely accounts for many differences in hospitalized patients and the care provided. Furthermore, there were no differences in other SOI measures across hospitals.

This study presents an analysis using administrative data to evaluate practice variation in pediatric critical care around the use of dexmedetomidine and is subject to the standard limitations of administrative data (28). This analysis relies on revenue data specifically the CTC system, which reflects care that was billed not necessarily administered. This analysis focused on evaluating the hospital-level rather than the patient-level care patterns. While this may limit its ability to be applied to a particular patient, this study was intended to assess the effect of a general practice style on overall outcomes. There are also limitations with the APR DRG severity-adjustment process including many factors that influence LOS and hospital charges. There are physiologically based severity-adjustment models available, but none can be applied administratively across multiple hospitals.

Despite these limitations, PHIS is the only pediatric specific, multi-institutional dataset where investigators can begin to assess general practice patterns and the potential affects on outcomes. This study reflects current practice patterns. While limited to patients admitted in 2013, it included many patients discharged in 2014. This type of analysis adopts the framework specified by the Institute of Medicine for a Learning Health Care System. The wide practice variation around the use of dexmedetomidine in the PICU exists without clear benefits to outcomes as far as LOS, charges, and mortality warrants further study. Clinically, dexmedetomidine seems to be a useful sedative in the PICU, but unsupported physician preference should not drive utilization. This study suggests that further work should not be focused on outcomes like LOS and mortality but rather on more patient-centered outcomes, such as delirium or long-term neurodevelopment. While this study did not find increased charges associated with dexmedetomidine use, benefits in long-term outcomes may have cost benefits for the entire healthcare system. As dexmedetomidine comes off patent and its cost lowers (presumably utilization will increase), it will be critical to understand how to use this medication, the optimal patient selection, and the expected benefits.

## Conclusion

Dexmedetomidine utilization has increased sfold in PICU patients who are mechanically ventilated, but continues to have 15-fold variation in practice with some hospitals using it rarely and others using it in over 60% of patients. There does not appear to be a significant difference in overall severity-adjusted LOS or hospital charges between hospitals that use dexmedetomidine frequently as compared to those that do not. Further work should explore the other potential outcome benefits (such as delirium or neurodevelopmental affects) of using dexmedetomidine in invasively ventilated patients.

## Author Contributions

Dr. BB wrote the first draft of the manuscript and all the authors have approved the final version.

## Conflict of Interest Statement

The authors declare that the research was conducted in the absence of any commercial or financial relationships that could be construed as a potential conflict of interest.

## Supplementary Material

Data Sheet S1 **Listing and explanation of CTC, APR DRGs, and PHIS flags**.

## Abbreviations

APR DRG, all-patient refined diagnosis related group; CHA, Children’s Hospital Association; CTC, clinical transaction classification; IQR, interquartile range; LOS, length of stay; PHIS, pediatric health information system; PICU, Pediatric Intensive Care Unit; RACHS-1, risk adjustment in congenital heart surgery, version 1; SOI, APR DRG severity of illness.

## References

[1] Chrysostomou C Morell VO Wearden P Sanchez-de-Toledo J Jooste EH Beerman L. Dexmedetomidine: therapeutic use for the termination of reentrant supraventricular tachycardia. Congenit Heart Dis (2013) 8(1):48–56. 10.1111/j.1747-0803.2012.00669.x 22613357

[2] Tobias JD Gupta P Naguib A Yates AR. Dexmedetomidine: applications for the pediatric patient with congenital heart disease. Pediatr Cardiol (2011) 32(8):1075–87. 10.1007/s00246-011-0092-8 21909772

[3] Czaja AS Zimmerman JJ. The use of dexmedetomidine in critically ill children. Pediatr Crit Care Med (2009) 10(3):381–6. 10.1097/PCC.0b013e3181a3191f 19325505

[4] Buck ML Willson DF Use of dexmedetomidine in the pediatric intensive care unit. Pharmacotherapy (2008) 28(1):51–7. 10.1592/phco.28.1.51 18154474

[5] Carroll CL Krieger D Campbell M Fisher DG Comeau LL Zucker AR. Use of dexmedetomidine for sedation of children hospitalized in the intensive care unit. J Hosp Med (2008) 3(2):142–7. 10.1002/jhm.282 18438790

[6] Tobias JD. Dexmedetomidine: applications in pediatric critical care and pediatric anesthesiology. Pediatr Crit Care Med (2007) 8(2):115–31. 10.1097/01.PCC.0000257100.31779.41 17273114

[7] Chrysostomou C Di Filippo S Manrique A-M Schmitt CG Orr RA Casta A Use of dexmedetomidine in children after cardiac and thoracic surgery. Pediatr Crit Care Med (2006) 7(2):126–31. 10.1097/01.PCC.0000200967.76996.07 16446599

[8] Aydogan MS Korkmaz MF Ozgul U Erdogan MA Yucel A Karaman A Pain, fentanyl consumption, and delirium in adolescents after scoliosis surgery: dexmedetomidine vs midazolam. Paediatr Anaesth (2013) 23(5):446–52. 10.1111/pan.12128 23448434

[9] Fagin A Palmieri T Greenhalgh D Sen S. A comparison of dexmedetomidine and midazolam for sedation in severe pediatric burn injury. J Burn Care Res (2012) 33(6):759–63. 10.1097/BCR.0b013e318254d48e 23147214

[10] Gupta P Whiteside W Sabati A Tesoro TM Gossett JM Tobias JD Safety and efficacy of prolonged dexmedetomidine use in critically ill children with heart disease. Pediatr Crit Care Med (2012) 13(6):660–6. 10.1097/PCC.0b013e318253c7f1 22791093

[11] RED BOOK Online^®^. Thomson Micromedex. Greenwood Village, CO: Thomson Reuters (2013).

[12] Committee on the Learning Health Care System in America, Institute of Medicine Smith M Saunders R Stuckhardt L McGinnis M Best Care at Lower Cost: The Path to Continuously Learning Health Care in America. Washington, DC: National Academies Press (2013). 24901184

[13] Children’s Hospital Association. Data Source: Pediatric Health Information System (PHIS) January 2007 to December 2013. Overland Park, KS: Children’s Hospital Association (2015).

[14] Feudtner C Feinstein JA Zhong W Hall M Dai D. Pediatric complex chronic conditions classification system version 2: updated for ICD-10 and complex medical technology dependence and transplantation. BMC Pediatr (2014) 14(1):1–7. 10.1186/1471-2431-14-199 25102958 PMC4134331

[15] Jenkins KJ Gauvreau K Newburger JW Spray TL Moller JH Iezzoni LI. Consensus-based method for risk adjustment for surgery for congenital heart disease. J Thorac Cardiovasc Surg (2002) 123(1):110–8. 10.1067/mtc.2002.119064 11782764

[16] Averill R McCullough EC Goldfield N Hughes JS Bonazelli J Bentley L 3M APR DRG Classification System. 31st ed Wallingford, CT: Agency for Healthcare Research and Quality (2013). p. 1–98. Report No.: GRP-041.

[17] Miller RL Gebremariam A Odetola FO. Pediatric high-impact conditions in the United States: retrospective analysis of hospitalizations and associated resource use. BMC Pediatr (2012) 12:61. 10.1186/1471-2431-12-61 22681875 PMC3502249

[18] Slonim AD Khandelwal S He J Hall M Stockwell DC Turenne WM Characteristics associated with pediatric inpatient death. Pediatrics (2010) 125(6):1208–16. 10.1542/peds.2009-1451 20457682 PMC3033561

[19] Baram D Daroowalla F Garcia R Zhang G Chen JJ Healy E Use of the all patient refined-diagnosis related group (APR-DRG) risk of mortality score as a severity adjustor in the medical ICU. Clin Med Circ Respirat Pulm Med (2008) 2:19–25. 21157518 10.4137/ccrpm.s544 PMC2990229

[20] Slonim AD Marcin JP Turenne W Hall M Joseph JG. Pediatric patient safety events during hospitalization: approaches to accounting for institution-level effects. Health Serv Res (2007) 42(6 Pt 1):2275–93. 10.1111/j.1475-6773.2007.00729.x 17995566 PMC2151391

[21] Benneyworth BD Gebremariam A Clark SJ Shanley TP Davis MM. Inpatient health care utilization for children dependent on long-term mechanical ventilation. Pediatrics (2011) 127(6):e1533–41. 10.1542/peds.2010-2026 21576303 PMC3103275

[22] Wennberg JE. Practice variation: implications for our health care system. Manag Care (2004) 13:3–7. 15493217

[23] Lopez AM Tilford JM Anand KJS Jo C-H Green JW Aitken ME Variation in pediatric intensive care therapies and outcomes by race, gender, and insurance status. Pediatr Crit Care Med (2006) 7(1):2–6. 10.1097/01.PCC.0000192319.55850.81 16395066

[24] Hartman ME McCrory DC Schulman SR. Efficacy of sedation regimens to facilitate mechanical ventilation in the pediatric intensive care unit: a systematic review. Pediatr Crit Care Med (2009) 10(2):246–55. 10.1097/PCC.0b013e31819a3bb9 19188867

[25] Weinberger M Oddone EZ Henderson WG. Does increased access to primary care reduce hospital readmissions? Veterans affairs cooperative study group on primary care and hospital readmission. N Engl J Med (1996) 334(22):1441–7. 10.1056/NEJM199605303342206 8618584

[26] Scales DC Laupacis A Health technology assessment in critical care. Intensive Care Med (2007) 33(12):2183–91. 10.1007/s00134-007-0909-3 17952404

[27] Garland A Shaman Z Baron J Connors AFJ. Physician-attributable differences in intensive care unit costs: a single-center study. Am J Respir Crit Care Med (2006) 174(11):1206–10. 10.1164/rccm.200511-1810OC 16973977

[28] Bennett TD Spaeder MC Matos REI Watson RS Typpo KV Khemani RG Existing data analysis in pediatric critical care research. Front Pediatr (2014) 2:79. 10.3389/fped.2014.00079 25121079 PMC4114296

