# Supplementary material for: Retrospective Evaluation of the Epidemiology and Practice Variation of Dexmedetomidine Use in Invasively Ventilated Pediatric Intensive Care Admissions, 2007–2013
Source: Front Pediatr. 2015 Dec 16;3:109. doi: 10.3389/fped.2015.00109 (PMC4679909; doi:10.3389/fped.2015.00109)
Supplement: Data Sheet S1 — Listing and explanation of CTC, APR DRGs, and PHIS flags. [file data_sheet_1.docx]

**Supplemental Content, eMethods:**

CTC, APR DRG, and ICD-9-CM codes for various categories

Intensive Care Unit Flag:

- CTC codes: 600605, 600620, 600625, 600640, 600645, 600650, 600655, 600699 (with a charge method of 1, 2, or 10-19)

Mechanical Ventilation:

- CTC codes: 521166 or 521169

Neonatal Care Exclusion:

- CTC codes: 600520 (charge method of 1, 2, or 10-19)
- APR DRGs: 580, 581, 583, 588, 589, 591, 593, 602, 603, 607, 608, 609, 611, 612, 613, 614, 621, 622, 623, 625, 626, 631, 633, 634, 636, 639, 640, 863

Cardiac surgical patients:

- APR DRGs: 2, 160, 161, 162, 163, 165, 166, 167, 170, 171, 174, 175, 176, 177, 190, 191, 192, 194, 196, 198, 200, 201, 205, 206, 630

Neurocritical care:

- APR DRGs: 20, 21, 22, 23, 26, 40, 42, 43, 44, 45, 46, 47, 48, 52, 53, 54, 55, 56, 57, 58, 204, 910

Prolonged Mechanical Ventilation:

- APR DRGs: 4, 5, 130
